# Do letters about conspiracy belief studies greatly exaggerate? A reply to Sutton and Douglas

**DOI:** 10.1017/S0033291720002913

**Published:** 2020-07-30

**Authors:** Daniel Freeman, Felicity Waite, Laina Rosebrock, Ariane Petit, Emily Bold, Sophie Mulhall, Lydia Carr, Ashley-Louise Teale, Lucy Jenner, Anna East, Chiara Causier, Jessica C. Bird, Sinéad Lambe

**Affiliations:** Psychiatry, Oxford University, UK

In the design of our study about coronavirus conspiracy beliefs and the consequences for adherence to social distancing guidelines (Freeman et al., 2020), we thought very carefully about the content of questionnaire items, and their associated scales, in order to test the primary hypothesis. We chose to develop item content that was unambiguous, extreme, and false (e.g. ‘Jews have created the virus to collapse the economy for financial gain’). We selected a response scale to assess any degree of endorsement (do not agree, agree a little, agree moderately, agree a lot, agree completely) in a manner easily understandable for participants and simple to interpret. Respondents were presented with stark beliefs and a clear decision to make about endorsement. Hence, we could test whether any countenance of the extreme beliefs − which might include a degree of acquiescence, though there was no ambiguity in the statements being endorsed − affects adherence to social distancing guidelines. It is a study about how belief may drive action and *any* belief in an obvious conspiracy theory might be socially damaging. It should not be forgotten that there was a very simple, low cognitive load option for responding to the extreme beliefs: ‘do not agree’. There is also evidence that online surveys can be resistant to demand effects (Mummolo & Peterson, 2019). The measurement method for conspiracy beliefs was grounded in clinical studies assessing delusions, in which a single dimension is isolated of degree of conviction in unfounded beliefs [from do not believe (0%) to completely believe (100%)]. Deliberately avoided is a single ‘completely disbelieve’ (−100%) to ‘completely believe’ (100%) scale. This avoidance is partly because of difficulties in interpreting such dimensions of disagreement and partly because of empirical evidence that shows that the degree to which an individual believes a delusional belief is separate (to an extent) from the degree to which he or she thinks that they could be mistaken (So et al., 2012).

Sutton and Douglas (2020) asked a convenience sample of 750 people to complete a small number of our conspiracy belief questions but using different rating scales. We wish to note just three simple points in response. First, their result will surprise no one: using different rating scales results in a (somewhat) different pattern of answers. It does not identify which scale might be best. Second, the letter writers have overlooked the basic research design principle that items and their corresponding scales are chosen for the particular purpose of a study. A consequence is that they have missed a genuinely interesting methodological question: do different rating scales have differential sensitivity in assessing whether conspiracy beliefs affect adherence to social distancing guidelines? Finally, the letter writers omit consideration of the significant limitations of the scales they advocate. They think that there is a single continuum between strong disagreement and strong agreement and hence that simply adding disagree responses and a ‘neither agree nor disagree’ response solves issues of scaling – this is mistaken on all three counts. Degrees of agreement and disagreement are obviously negatively associated but typically they are not genuine opposites of a single dimension and it creates difficulties in interpretation when they are treated as so (Saris, Krosnick, Revilla, & Shae, 2010). The interpretative problems of introducing disagree options to a linear agree scale can be acutely seen if one pauses for a moment to consider our conspiracy theory study. For example, it would be plausible to think that a respondent who only ‘disagrees a little’ with the item ‘Jews have created the virus to collapse the economy for financial gain’ might also ‘agree a little’ with the extreme belief, but he or she would only be able to select one option. Sutton and Douglas add further imprecision with their use of the notoriously ambiguous midpoint response of ‘neither agree nor disagree’, known to be selected for many different reasons by respondents (Kulas & Stachowski, 2013). If we were to finesse our scale, we would consider adding a ‘Don't know’ response option, although this too is not without complications since there is a decision to make about how to treat such responses in analyses.

No questionnaires are perfect^1^, but our choice of item content and associated scaling was conceptually precise, easy to understand, and easy to interpret. If Sutton and Douglas are as fixed on introducing disagreement as they seem, then they should have added a second rating scale for disagreement for each item. Their letter concludes with an age-old lament about press releases purportedly stripping research coverage of nuance and caveats and introducing sensationalism; we hope such injudicious traits are equally guarded against in journal letters.

## Note

1 Except ours.
